# Statins as an Adjunctive Antithrombotic Agent in Thrombotic Antiphospholipid Syndrome: Mechanisms and Clinical Implications

**DOI:** 10.3390/cells14050353

**Published:** 2025-02-28

**Authors:** Tommaso Bucci, Danilo Menichelli, Ilaria Maria Palumbo, Daniele Pastori, Paul R. J. Ames, Gregory Y. H. Lip, Pasquale Pignatelli

**Affiliations:** 1 Liverpool Centre for Cardiovascular Science, University of Liverpool, Liverpool John Moores University and Liverpool and Heart and Chest Hospital, Liverpool, L7 8TX, UK; tommaso.bucci@liverpool.ac.uk (T.B.); daniele.pastori@uniroma1.it (D.P.); gregory.lip@liverpool.ac.uk (G.Y.H.L.); 2 Department of Clinical Internal, Anaesthesiologic and Cardiovascular Sciences, Sapienza University of Rome, 00185 Rome, Italy; danilo.menichelli@uniroma1.it (D.M.); ilariamaria.palumbo@uniroma1.it (I.M.P.); 3 Department of General and Specialized Surgery “Paride Stefanini”, Sapienza University of Rome, 00185 Rome, Italy; 4 IRCCS Neuromed, Località Camerelle, 86077 Pozzilli, Italy; 5 Immune Response and Vascular Disease, iNOVA, 4Health, Nova Medical School, Nova University Lisbon, 1099-085 Lisbon, Portugal; paul.ames2@nhs.scot; 6 Department of Haematology, Dumfries Royal Infirmary, Cargenbridge, Dumfries DG2 8RX, UK; 7 Danish Centre for Health Services Research, Department of Clinical Medicine, Aalborg University, 9220 Aalborg, Denmark; 8 Department of Cardiology, Lipidology and Internal Medicine, Medical University of Bialystok, 15-089 Bialystok, Poland

**Keywords:** antiphospholipid syndrome, statins, thrombosis, mechanism

## Abstract

The thrombotic physiopathology of antiphospholipid syndrome (APS) is complex, heterogeneous, and dynamic. While venous thromboembolism (VTE) is the most common initial presentation, arterial thrombotic events (ATE) become more frequent in advanced stages and are associated with high morbidity and mortality. Despite the use of oral anticoagulants (OACs), thrombotic APS remains associated with a high risk of recurrent thrombosis. Given their potential antithrombotic effects capable of reducing the risk of both VTE and ATE, statins have been proposed as an adjunctive therapy to OACs for patients with APS and recurrent thrombosis. However, this recommendation is primarily based on studies not specifically conducted in APS populations, with only preclinical data or evidence from retrospective observational studies available from APS patients cohorts. For these reasons, this narrative review aims to synthesise the studies evaluating the potential antithrombotic effects of statins in patients with APS, highlighting the progress made and identifying areas for future research.

## 1. Introduction

Antiphospholipid syndrome (APS) is an acquired thrombotic disease, characterised by arterial (ATE) and venous thromboembolism (VTE) and/or pregnancy morbidity in the presence of persistent positivity of antiphospholipid antibodies (aPL), including anti-cardiolipin (aCL) IgG/IgM, anti β2 glycoprotein I (aβ2GPI) IgG/IgM, and lupus anticoagulant (LAC) [1].

Given the relative rarity of this clinical condition, the changes in the definition of the APS classification criteria over time, the difficulties in the standardisation of aPL measurements, and the differences in laboratory cutoffs, the precise prevalence of APS in the general population is still unclear. Data from the United States [2], Italy [3], and Spain [4] estimate APS prevalence at about 17–50 cases per 100,000 persons. APS incidence is estimated to be between 2 and 5 new cases per 100,000 persons per year [5].

APS can be classified as secondary APS when associated with other autoimmune diseases (i.e., systemic lupus erythematosus, rheumatoid arthritis, systemic sclerosis, Sjögren syndrome, polymyositis, or dermatomyositis) or primary APS when isolated [6]. APS can also be classified considering the antibody profile (single, double, or triple positivity) or based on the prevalent clinical manifestation (i.e., thrombotic or obstetric) [6].

This narrative review focuses exclusively on the thrombotic manifestations of APS, with the aim of providing evidence supporting the use of statins as antithrombotic agents in conjunction with oral anticoagulants (OACs) to mitigate the risk of recurrent thrombosis.

Therefore, we initially detail the specific thrombogenic pathways of APS, and then we focus on the pharmacological effects of statins on these pathways. Additionally, we review the principal studies examining the impact of statins on thrombotic and inflammatory markers involved in the thrombotic process in patients with APS. We also summarise clinical evidence demonstrating the protective role of statins in reducing the risk of cardiovascular events, including studies on patients without autoimmune disease and all studies conducted on patients with thrombotic APS to date.

## 2. Thrombotic Manifestations of APS

Previous studies have shown that approximately 10% of VTE cases may be associated with underlying APS [7]. Moreover, VTE is the most frequent initial thrombotic manifestation, occurring in nearly 40% of patients with APS, with deep vein thrombosis of the lower limbs—whether accompanied by pulmonary embolism or not—being the most common presentation [8]. Other venous sites may also be affected, including those in the upper arms, cerebral sinuses, and splanchnic circulation [9].

Regarding arterial involvement, the most common type of ATE in patients with APS is ischaemic stroke [10]. It has been estimated that approximately 20% of APS patients experience an ischaemic stroke, and 11.1% have a transient ischemic attack as the initial thrombotic manifestation [8]. Less commonly, patients with APS present with acute myocardial infarction or thrombosis of the peripheral arterial system as the initial manifestation [8,11,12]. However, although ATE is less common as an initial manifestation of APS compared to VTE, it represents the most frequent type of thrombotic recurrence in patients already on OACs and is associated with the highest risk of morbidity and mortality [13].

In rare cases, APS can present with thrombotic manifestations involving the microcirculatory system, potentially affecting several vascular beds, including those of the kidneys, retina, skin, and brain [14]. Widespread microvascular thrombosis involving multiple organs characterises catastrophic APS, a life-threatening condition associated with multiorgan failure and high mortality risk [15].

## 3. Thrombogenesis in Patients with Antiphospholipid Syndrome

aPL do not directly bind to membrane or plasma phospholipids but instead interact with them by binding to plasma proteins, among which β2GPI is the most well-characterised. β2GPI is a 43 kDa plasma protein widely expressed on various cell types, with important functions that modulate immunological and thrombotic responses [16,17]. β2GPI exists in two distinct forms: a closed, circular conformation, where its domains are tightly folded, preventing the exposure of binding sites to phospholipids and other cellular receptors; and an open conformation that can interact with anionic surfaces—a process crucial for thrombosis initiation [18]. Changes in β2GPI conformation are related to the presence of a pro-oxidant state characterised by the presence of a large amount of reactive oxidative species (ROS) [19], which may disrupt disulfide bonds, resulting in the unfolding of β2GPI and the exposure of hidden epitopes. This conformational shift to an open form sustains the production of aβ2GPI antibodies in predisposed individuals and triggers pro-thrombotic processes [20].

**Figure 1:**
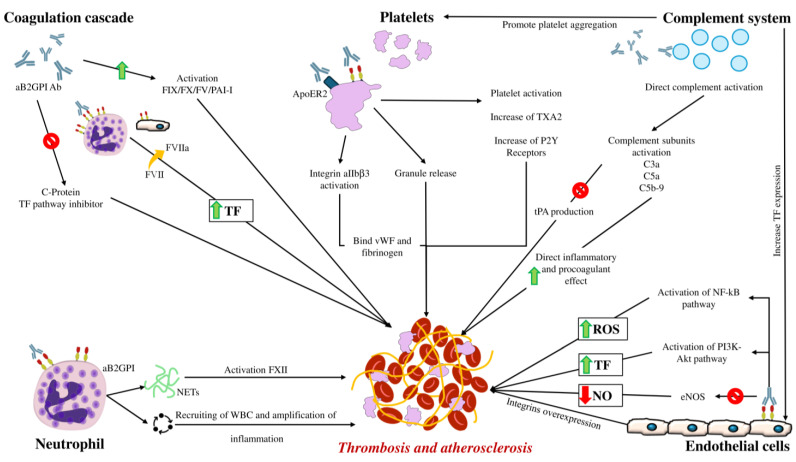
Mechanisms of thrombogenesis in antiphospholipid syndrome. aB2GPI Ab: anti-beta2glycoprotein I antibodies, eNOS: endothelial nitric oxide synthase, FIX/FX/FV/PAI-I: anticoagulation factor IX/X/V/plasminogen activator inhibitor -I, FVII/FVIIa: anticoagulation factor VII/VII activated, FXII: anticoagulation factor XII, NETs: neutrophil extracellular traps, NF-kB: nuclear factor k B, NO: nitric oxide, ox-LDL: oxidised low-density lipoprotein, ROS: reactive oxygen species, TF: tissue factor, tPA: tissue plasminogen activator TXA2: thromboxane A2, vWF: Von Willebrand factor, WBC: white blood cells.

Although the major role of the APS-related thrombotic process is performed through the interaction of antibodies with endothelial cells, the coagulation cascade, platelets, neutrophils, and the complement system also play roles (as shown in Figure 1).

### 3.1. The Role of Direct Activation of the Coagulation Cascade in Thrombosis

aβ2GPI antibodies bind to β2GPI expressed on the surface of endothelial cells, monocytes, and macrophages, inducing cellular activation and upregulating tissue factor (TF), a key mediator of thrombosis [21]. Once expressed on the cell surface, TF may facilitate FVII autoactivation (FVIIa). The TF-FVIIa complex then activates factor X, leading to thrombin generation. Also, aβ2GPI antibodies bind to activated factor IX, impairing antithrombin regulation and promoting the downstream activation of factor X and thrombin production [22]. These antibodies also directly enhance activated factor X activity, further driving thrombin generation and clot formation [23]. Additionally, aβ2GPI antibodies interact with factor V, increasing pro-thrombinase complex activity and accelerating thrombin production. They can also bind to factor XI, activating it and amplifying the intrinsic coagulation pathway [24].

Furthermore, aβ2GPI antibodies not only activate the clotting cascade but also impair natural anticoagulant systems. For instance, they interfere with the anticoagulant function of activated protein C, leading to the ineffective inactivation of activated factors V and VIII [25]. Additionally, they increase plasminogen activator inhibitor-1 (PAI-1) levels, which enhances clot formation and suppresses fibrinolytic pathways [26]. Furthermore, aβ2GPI antibodies may inhibit the TF pathway inhibitor (TFPI), thereby amplifying the activity of the TF-FVIIa complex and further propagating the coagulation cascade [27,28].

### 3.2. The Role of Platelet Activation in Thrombosis

One of the most studied mechanisms by which aβ2GPI antibodies activate platelets is through their interaction with the apolipoprotein E receptor 2 (ApoER2) expressed on the platelet surface. The binding of aβ2GPI antibody/β2GPI complexes to ApoER2 increases platelet aggregation, thromboxane A2 (TXA2) production, granule release, and integrin αIIbβ3 activation [29]. These effects are closely related to the availability of GPIbα on the platelet surface, which binds the aβ2GPI antibody/β2GPI/ApoER2 complex and induces the phosphorylation of p38 mitogen-activated protein kinase (MAPK), a key step in platelet activation [29]. The phosphorylation of p38 MAPK increases intracellular ROS production via nicotinamide adenine dinucleotide phosphate (NADPH) oxidase, which in turn activates cyclooxygenase-2 (COX-2), leading to the production of TXA2 and prostaglandins (PGE2 and PGI2) [30]. These mediators contribute to platelet activation by inducing conformational changes, activating P2Y purinergic receptors for adenosine diphosphate (ADP), increasing intracellular calcium concentration, inducing granule release, and stimulating αIIbβ3 integrin activation, which facilitates platelet aggregation by interacting with von Willebrand factor (vWF) and fibrinogen [31,32]. Furthermore, platelet cytoskeleton remodelling, driven by small GTPases, such as RhoA and Rac1, supports the spread and stabilisation of platelet aggregates, contributing to the formation of a stable platelet plug essential for haemostasis [33].

Another important pathway for platelet activation by aβ2GPI antibodies is through interaction with toll-like receptors 2 (TLR2) and 4 (TLR4). Binding to TLR2 activates platelets by enhancing phosphatidylinositol 3-kinase (PI3K) signalling [34], while binding to TLR4 triggers the activation of the NF-κB and MAPK pathways, leading to inflammatory responses that contribute to APS-related thrombosis [35]. aβ2GPI antibodies also bind to Fcγ receptors on platelets, activating Syn (stress-activated protein kinase-interacting protein) and further promoting platelet aggregation, and granule secretion upregulating the mTORC2/Akt pathway [36]. Additionally, they can facilitate phosphatidylserine exposure via the annexin V pathway, enhancing coagulation, and induce the release of platelet-derived micro vesicles that carry activated coagulation factors, promoting a systemic pro-thrombotic environment in APS patients [37].

### 3.3. The Role of Neutrophil Activation in Thrombosis

In recent years, growing evidence has suggested that neutrophils play an important role in thrombosis in patients with APS. Neutrophils employ various effector mechanisms, including ROS production, phagocytosis, the release of proteases, and the formation of extracellular traps (NETs), to combat infections, eliminate pathogens, and mitigate inflammatory damage to surrounding vascular tissues [38]. NETs have been associated with various pro-thrombotic states and hypercoagulability, including acute coronary syndromes, atrial fibrillation, cancer, and infections such as COVID-19 [39,40,41,42].

In the pathogenesis of thrombotic APS, the pro-thrombotic activity of NETs appears to play a significant role. NETs are composed of extracellular DNA lattices intertwined with citrullinated histones and neutrophil proteases, which are released by neutrophils to immobilise and facilitate the elimination of microorganisms [43]. Elevated levels of NETs have been observed in various clinical conditions associated with an increased risk of thrombosis, including APS [44,45]. Indeed, the interaction of aβ2GPI antibodies with β2GPI expressed on the neutrophil surface can initiate an activation signal, stimulating and amplifying NET formation [46]. These NETs, in turn, directly bind to factor XII, activating the intrinsic coagulation pathway [47]. They also interact with fibrinogen to promote fibrin deposition and clot stabilisation [48], stimulate the NLRP3 inflammasome, a protein complex that plays a crucial role in the maturation of Interleukin (IL)-1β and IL-18 [49], and interact with TLR-4 on platelets, favouring their activation and aggregation. Additionally, they inhibit fibrinolysis [50] and perpetuate the pro-inflammatory response by recruiting other immune cells, such as monocytes and macrophages, thereby sustaining this pro-inflammatory vicious cycle [38]. Furthermore, NETs can activate the complement system, contributing to platelet and endothelial activation while impairing the resolution of inflammation and the activity of fibrinolytic proteins, making it more difficult to break down formed thrombi [38].

### 3.4. The Role of Complement Activation in Thrombosis

An additional mechanism involved in the pathophysiology of thrombosis in patients with APS is the activation of the complement system [51]. The complement system is an enzymatic cascade of proteins that promotes inflammation and defends against microorganisms [52]. It includes three pathways: the classical pathway (triggered by antigen–antibody complexes), the lectin pathway (activated by pattern-recognition molecules binding to pathogens), and the alternative pathway (initiated by the spontaneous hydrolysis of C3) [52]. These pathways converge to form C3 and C5 convertases, which cleave C3 into C3b and C3a, and C5 into C5b and C5a. C5b forms the membrane attack complex (MAC), causing cell lysis, while C3a and C5a release pro-inflammatory cytokines [52]. In animal models, it has been shown that the activation of C3 and C5 is a necessary step for aβ2GPI antibody-mediated thrombosis [53]. Indeed, aβ2GPI antibodies can induce complement activation, potentially activating all three complement pathways. This results in the production of large amounts of end products (C3a, C5a, C5b-9), which have important procoagulant and pro-inflammatory effects on monocytes [54]. Additionally, it has been shown that C5a binds to C5a receptors on platelets, promoting platelet activation, aggregation, and thrombus formation [55]. Complement activation also induces endothelial cell activation, increasing the expression of pro-thrombotic molecules, such as TF. Moreover, C3a and C5a contribute to endothelial dysfunction by promoting vascular permeability, leukocyte recruitment, and platelet aggregation, all of which help establish a pro-thrombotic state [56]. The products of complement activation also perpetuate a feedback loop of inflammation and thrombosis, amplifying the release of pro-inflammatory cytokines and sustaining the pro-thrombotic environment [57]. Finally, C5b-9 impairs fibrinolysis by sequestering plasminogen and inhibiting tissue plasminogen activator, thus contributing to clot persistence [58].

### 3.5. The Role of Endothelial Cell Activation in Thrombosis and Atherosclerosis

Although aβ2GPI antibodies can induce thrombosis through direct activation of the coagulation cascade, as well as through interactions with platelets, immune cells, and complement, the cornerstone of the APS-related thrombotic process is the interaction of aβ2GPI antibodies with endothelial cells [59]. Endothelial cells are highly specialised cells that line the inner surface of the circulatory system, where they modulate vascular tone, coagulation activation, and the recruitment of immune cells in response to various stimuli and clinical or pathological conditions [60]. Most of these fundamental functions are regulated by the production of nitric oxide (NO) through endothelial nitric oxide synthase (eNOS) [61]. In physiological conditions, the production of NO helps establish an anti-inflammatory and anticoagulant environment. Conversely, in pathological conditions, particularly following vascular damage, NO availability decreases, resulting in a pro-inflammatory phenotype characterised by vasoconstriction, activation of the coagulation cascade, and leukocyte recruitment—processes essential for repairing vascular damage [62].

Animal models of APS have shown that aβ2GPI antibodies can interact with β2GPI expressed on endothelial cells, stimulating a pro-inflammatory and pro-coagulant response akin to that observed in the presence of vascular damage. Specifically, aβ2GPI antibodies activate the NF-κB pathway, which increases the expression of integrins and selectins, thereby facilitating leukocyte adhesion and diapedesis [63]. They can also interfere with eNOS, leading to its uncoupling, which reduces NO production and increases the release of ROS, including superoxide anions (O₂⁻), hydrogen peroxide (H₂O₂), and hydroxyl radicals (OH⁻) [64]. These ROS perpetuate pro-inflammatory effects, further reducing NO bioavailability and promoting the oxidation of lipids and proteins, thus contributing to vascular damage. Indeed, aβ2GPI antibodies can activate intracellular pathways, such as p38 MAPK and ERK1/2 (extracellular signal-regulated kinase 1/2), which contribute to the increased activity of oxidative enzymes, including NADPH oxidase, myeloperoxidase, xanthine oxidase, and components of the mitochondrial respiratory chain [65]. This is accompanied by a reduction in the activity of antioxidant enzymes, such as superoxide dismutase, glutathione peroxidase, haemoxygenase, thioredoxin peroxidase/peroxiredoxin, catalase, and paraoxonase [65]. Moreover, aβ2GPI antibodies can initiate the PI3K-Akt pathway, increasing the expression of TF and leading to the reduced survival of endothelial cells [66]. Additionally, the activation of TLR-4 and the Janus kinase/signal transducers and activators of the transcription (JAK/STAT) pathway leads to the increased production of pro-inflammatory and pro-coagulant cytokines, such as IL-6 and tumour necrosis factor α (TNF-α) [67]. Lastly, aβ2GPI antibodies contribute to the accumulation of misfolded or unfolded proteins in the endoplasmic reticulum (ER), inducing ER stress [68]. This, in turn, activates the unfolded protein response (UPR), which involves the activation of inositol-requiring enzyme 1 (IRE1), protein kinase RNA-like ER kinase (PERK), and activating transcription factor 6 (ATF6) [69]. These pathways further promote endothelial dysfunction and contribute to a systemic state of inflammation and thrombosis.

All the mechanisms triggered by aβ2GPI antibodies induce thrombosis not only by directly activating the coagulation cascade but also through the promotion of atherosclerosis [70]. Indeed, the presence of this pro-inflammatory state is responsible for the onset of endothelial dysfunction, which favours the formation of lipid plaques in arterial vessels and the production of oxidised low-density lipoproteins (oxLDL), which are highly pro-atherogenic and recognised by aβ2GPI antibodies [71]. The ingestion of the oxLDL-aβ2GPI antibody complexes by macrophages leads to the formation of foam cells, which are unable to eliminate these complexes and eventually undergo apoptosis within the atherosclerotic plaque, triggering an uncontrolled inflammatory response that attracts other inflammatory cells into the vessel wall, responsible first for plaque growth, then for the erosion of the fibrous cap, and ultimately for plaque rupture, exposing the extracellular matrix to the bloodstream and activating the coagulation cascade [72]. A summary of the thrombotic mechanisms involved in APS is reported in Figure 1.

## 4. Antithrombotic Strategies in Patients with APS and Recurrent Thrombosis

To date, OACs remain the primary approach for secondary thromboprophylaxis in APS patients, with vitamin K antagonists (VKAs) as the cornerstone of treatment, while non-vitamin K antagonist oral anticoagulants (NOACs) are a debated option, recommended only for patients with a low-risk aPL profile or significant contraindications to VKA therapy [73,74].

For patients with APS who experience a first thrombotic event, international guidelines recommend initiating moderate-intensity VKA therapy, targeting an international normalised ratio (INR) of 2.0 to 3.0, with heparin bridging [73]. However, despite this, patients with APS still face a residual risk of thrombosis, ranging between 3% and 25% [75,76,77]. For those who develop recurrent thrombosis while on OACs, various strategies have been proposed.

One approach is to increase the INR target from 2.0–3.0 (moderate intensity) to 3.0–4.0 (high intensity) [73]. This indication has been mainly derived from two randomised clinical trials (RCTs) investigating the safety and efficacy of high-intensity treatment compared to moderate-intensity treatment. The first study, which included 114 patients with APS followed for a mean duration of 2.7 years, found no difference in the risk of recurrent thrombosis (hazard ratio [HR] of 3.1, 95% confidence interval [CI] of 0.6–15.0) or major bleeding (HR of 1.0, 95% CI of 0.2–4.8) between the two approaches [78]. These findings were corroborated by the second RCT involving 109 patients with APS followed for 3.2 years. This study also found no statistically significant differences in the risk of recurrent thrombosis (HR of 1.97, 95% CI of 0.49–7.89), while observing a non-statistically significant trend toward an increased risk of major bleeding (HR of 2.18, 95% CI of 0.92–5.15) in patients on high-intensity versus moderate-intensity treatment [79]. However, these studies showed the same efficacy for a high-intensity VKA regimen compared to a moderate-intensity regimen and did not provide any definitive answers about the best anticoagulant approach for patients who experience thrombotic recurrence while on OACs. Additionally, it should be noted that, in both studies, the lack of baseline stratification of APS patients by aPL titre (or profile) in the moderate- and high-intensity OAC treatment groups may have influenced the results.

The second approach is to add low-dose aspirin (LDA, 100–300 mg) to VKA therapy with moderate intensity [73]. However, this strategy is recommended only for those with a previous history of ATE or individuals at high risk, such as those with triple positivity (defined as the concurrent presence of aCL and aβ2GPI antibodies along with LAC positivity). The limited evidence available for this approach comes from a meta-analysis that extrapolated data from a broader cohort. This analysis included 21 patients (who experienced 7 thrombotic events) treated with OACs and LDA, compared to 13 patients (who experienced 10 thrombotic events) receiving VKA alone (risk ratio of 0.43, 95% CI of 0.22–0.85). Notably, the meta-analysis did not report an increased risk of bleeding, although this is likely due to low statistical power [80].

The third approach is to switch from VKA to low-molecular-weight heparin (LMWH). International guidelines suggest this as a potential option for patients who experience recurrent thrombosis despite achieving the target INR of 2–3 with VKA therapy [73]. In such cases, switching to LMWH might be considered, particularly if other options prove ineffective or are contraindicated. However, the long-term use of LMWH is often constrained by practical challenges, including the inconvenience of frequent subcutaneous injections, high financial costs, and the potential risk of bone fractures [81,82]. Moreover, despite its often transient course occurring only during the early administration of LMWH, heparin-induced thrombocytopenia may further reduce platelet count in patients with APS and persistent thrombocytopenia [83,84].

Hydroxychloroquine (HCQ) has also been proposed as a potential adjuvant therapy for patients with APS and recurrent thrombosis. HCQ inhibits phospholipase A2, blocking the release of arachidonic acid, which in turn decreases TXA and PGI production, exerting a significant antiplatelet effect [85]. Additionally, HCQ inhibits TF expression on monocytes and enhances NO availability in endothelial cells [86]. In a pilot open-label randomised trial investigating the efficacy of HCQ for thrombosis prevention, 50 PAPS patients were treated with standard care and HCQ, and another 50 PAPS patients were treated with standard care only. After a mean follow-up of 2.6 years, the authors found a significant reduced risk of thrombosis in HCQ users even after adjusting for confounders (HR of 0.09, 95% CI of 0.01–1.26) [87].

In addition to these options, the 2019 EULAR guidelines for APS management mention the use of statins as a potential adjuvant antithrombotic approach alongside VKAs in patients with recurrent thrombosis [73]. In patients with APS, statins represent a cornerstone therapy for primary and secondary thromboprophylaxis and play a critical role in their clinical management. However, the evidence supporting the EULAR recommendation primarily comes from studies conducted in patients with SLE or without autoimmune diseases, and only few pre-clinical and retrospective studies are available on APS.

## 5. Antithrombotic Effects of Statins

Statins are lipid-lowering drugs that inhibit cholesterol biosynthesis by downregulating hydroxymethylglutaryl-coenzyme A (HMG-CoA) reductase [88]. Beyond their lipid-lowering effects, which have also been demonstrated in autoimmune settings [89], statins play a significant role in inhibiting coagulation and clotting pathways in both arterial and venous circulation [90]. This antithrombotic effect arises from different mechanisms that counteract the coagulation process through both direct and indirect pathways. Therefore, statins may be potentially useful to address the heterogeneous thrombogenic pathways in APS.

By inhibiting HMG-CoA reductase, statins block the production of mevalonate, an essential precursor for the prenylation of the small GTPases RhoA and Rac1. Without prenylation, these proteins become less active, resulting in reduced nuclear translocation of NF-κB and decreased transcription of TF [91]. Notably, this effect seems to commence early during statin therapy: even after just three days of statin administration, a significant reduction in the pro-thrombin fragment F1+2—a marker of thrombin generation—and activated factor V can be observed [92,93]. Statins also downregulate the AP-1 transcription factor by modulating MAPKs, further reducing TF expression [94]. Additional direct antithrombotic effects include the inhibition of sterol regulatory element-binding proteins (SREBPs), which regulate the transcription of genes encoding coagulation factors, such as factor VII [95]. Furthermore, the suppression of isoprenoid production in hepatocytes leads to altered intracellular signalling and the decreased synthesis of factors VII and Va [96].

Moreover, statins can counteract platelet activation and aggregation, as evidenced by ex vivo studies on functional platelet aggregation and assessments of circulating soluble CD40L and P-selectin [97]. This inhibitory effect is primarily achieved through the downregulation of COX-1 and the upregulation of eNOS [98,99,100]. Additional mechanisms include the downregulation of NOX2 (the catalytic subunit of NADPH oxidase) and phospholipase A2, resulting in lower levels of PGE2 and TXA2 [101]. Furthermore, alterations in the lipid composition of platelet membranes reduce their flexibility and activation [102].

The inhibition of RhoA and Rac1 is particularly significant in mitigating neutrophil-related thrombosis. These GTPases play critical roles in neutrophil activation, chemotaxis, and degranulation [103]. Their inhibition leads to the reduced production of ROS, which subsequently decreases the release of NETs. Statins also inhibit the initiation of the complement cascade and MAC formation by decreasing ROS production and enhancing the expression of complement regulatory proteins, such as CD46, CD55, and CD59 [104,105].

**Figure 2:**
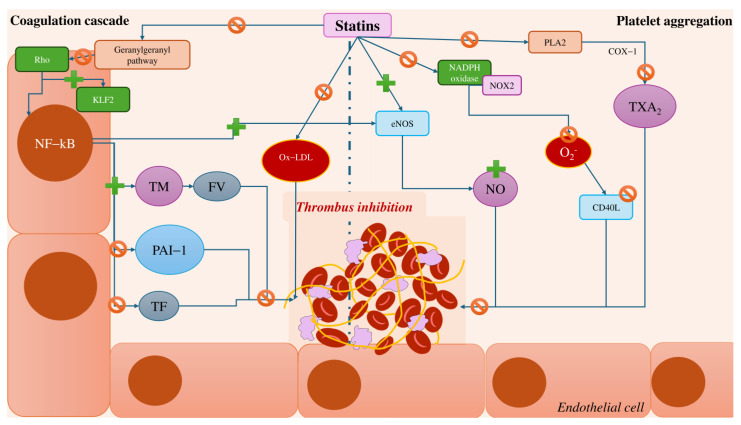
Summary of mechanisms involved in the antithrombotic property of statins. COX-1: cyclooxygenase-1, eNOS: endothelial nitric oxide synthase, FV: factor V, KLF2: Kruppel-like factor 2, NF-kB: nuclear factor k B, NO: nitric oxide, ox-LDL: oxidised low-density lipoprotein, PAI-1: plasminogen activator inhibitor-1, PLA2: phospholipase A2, TF: tissue factor, TM: thrombomodulin, TXA_2_: thromboxane A2.

Statins exert additional beneficial effects on endothelial cells by improving endothelial function and reducing inflammation, which indirectly influences thrombotic processes. They inhibit key signalling pathways, including NF-κB, PI3K/Akt, and TGF-β, resulting in lower levels of PAI-1 and enhanced fibrinolysis. By increasing NO bioavailability, statins promote vasodilation and reduce platelet activation and oxidative stress. Furthermore, statins decrease the expressions of adhesion molecules, such as ICAM-1 and VCAM-1, thereby reducing leukocyte adhesion and migration and mitigating inflammation. A summary of the antithrombotic mechanisms of statins is presented in Figure 2.

## 6. Clinical Evidence Supporting the Antithrombotic Effect of Statins in Patients Without Autoimmune Disease

Statins represent the cornerstone of secondary prophylaxis for several thrombotic diseases. In patients without autoimmune disease, statin treatment has been shown to be effective in reducing the risk of both ATE and VTE. Regarding the risk of ATE, a meta-analysis of 13 RCTs showed that early intensive statin therapy after acute coronary syndrome reduced the risk of death and cardiovascular events (HR of 0.81, 95% CI of 0.77–0.87), even after only 4 months of treatment [106]. This protective effect of statins was also confirmed by another meta-analysis of 13 RCTs, which showed a significantly reduced risk of major cardiovascular adverse events (odds ratio [OR] of 0.56, 95% CI of 0.44–0.71) in patients prescribed high-dose statins compared to those not prescribed statins or prescribed low-dose statins [107].

The protective effect of statins on ATE recurrence has also been confirmed for ischaemic cerebral events. In patients who were taking statins prior to an ischaemic stroke, less severe manifestations were reported (OR of 0.37, 95% CI of 0.19–0.74) [108]. In the SPARCL (Stroke Prevention by Aggressive Reduction in Cholesterol Levels) trial, patients with a recent stroke who were randomised to receive 80 mg of atorvastatin daily showed a 5-year absolute reduction of 2.2% in the risk of stroke recurrence in those treated with statins (HR of 0.84, 95% CI of 0.71–0.99) compared to those not treated with statins [109]. This protective effect was also observed in patients with atrial fibrillation who were on OACs. A retrospective study of 20,902 patients with ischaemic stroke, of whom 7500 (35.9%) received statins within 28 days of their stroke, found that statin use was associated with a lower 2-year risk of recurrent ischaemic stroke (HR of 0.45, 95% CI of 0.41–0.48), mortality (HR of 0.75, 95% CI of 0.66–0.84), intracranial haemorrhages (HR of 0.59, 95% CI of 0.47–0.72), acute myocardial infarction (HR of 0.35, 95% CI of 0.30–0.42), and hospital readmission (HR of 0.46, 95% CI of 0.42–0.50) [110].

Lastly, the protective effect of statins on ATE has also been shown in patients with peripheral artery disease. A recent systematic review and meta-analysis of 138,060 patients with peripheral artery disease, including 2 RCTs, 20 prospective studies, and 29 retrospective studies, found that only 35.1% of patients were treated with statins. Statins reduced the incidence of major adverse limb events by 30%, amputations by 35%, all-cause mortality by 39%, and ischaemic stroke by 28% [111]. Moreover, statin use was associated with improved survival, better limb salvage, and a lower risk of cardiovascular events, even after surgical or endovascular intervention, as well as with a mortality benefit following amputation [112,113].

The antithrombotic effect of statins may be useful not only for patients with ATE, but also, as shown by growing evidence, for patients with VTE. In the context of VTE, the antithrombotic effect of statins cannot be fully explained by the reduction in LDL levels alone, suggesting a more prominent role of the well-known anti-inflammatory pleiotropic effects of statins [114].

There has been only one RCT that has specifically evaluated a statin versus a placebo for the primary prevention of VTE: the JUPITER trial, which involved 17,802 participants with LDL levels of less than 130 mg/dL and high-sensitivity C-reactive protein levels of at least 2.0 mg/L. The participants were randomised to receive 20 mg per day of rosuvastatin or a placebo. During a median follow-up of 1.9 years, the authors found a statistically significant reduction in the risk of VTE in the rosuvastatin group compared to the placebo group (HR of 0.57, 95% CI of 0.37 to 0.86) [115]. However, a recent meta-analysis of 27 studies including 122,601 patients found that using statins for primary prevention may slightly reduce the incidence of VTE (OR of 0.86, 95% CI of 0.76–0.98), although this effect is likely too weak to be considered significant [116]. Thus, RCTs specifically designed to evaluate the potential benefits of statins in the primary prevention of VTE are still needed.

When considering VTE recurrences, more evidence is available. A prospective study of 432 patients (median age 65.5 years) followed for a median of 29.5 months after the discontinuation of anticoagulation found no association between statin use and recurrent VTE in patients with a first unprovoked event (HR of 1.02, 95% CI of 0.36–2.91) [117]. Conversely, a meta-analysis of eight observational studies found a reduced risk for recurrent VTE (relative risk [RR] of 0.73, 95% CI of 0.68–0.79) when comparing statin use with no use. The RRs for recurrent PE and DVT were 0.75 (95% CI of 0.58–0.96) and 0.66 (95% CI of 0.60–0.71), respectively [118]. This was further supported by another meta-analysis that included 23 RCTs with 118,464 participants, finding that the RR for VTE was 0.85 (95% CI of 0.73–0.99) when statin therapy was compared with placebo or no treatment. Rosuvastatin was associated with the lowest risk for VTE compared to other statins (HR of 0.57, 95% CI of 0.42–0.75) [119]. Moreover, these findings were confirmed by a national cohort study involving 44,430 patients with VTE, which found a reduced risk of recurrences in statin users (HR of 0.74, 95% CI of 0.68–0.80) [120], and by the COMMAND-VTE Registry, which included 3027 patients, 437 of whom were prescribed statins. The study found a significantly lower cumulative 3-year incidence of recurrent VTE in the statin group, even after adjusting for confounders (HR of 0.49, 95% CI of 0.30–0.81) [121]. The results are also supported by a recent meta-analysis of 45 RCTs, including more than 250,000 patients, which showed that both high-intensity (RR of 0.84, 95% CI of 0.70–1.02) and low-intensity statin monotherapy were associated with a positive trend toward VTE risk reduction (RR for high-intensity of 0.84, 95% CI of 0.70–1.02; RR for low-intensity of 0.89, 95% CI of 0.79–1.00) [122]. Additional information is expected from an ongoing RCT evaluating rosuvastatin for the prevention of recurrences and post-thrombotic syndrome (NCT04319627).

## 7. Evidence Supporting the Antithrombotic Effects of Statins in APS

Most the studies investigating the potential beneficial effects of statins on the thrombotic process mediated by aPL have come from preclinical research (Table 1). Meroni et al. conducted the first pioneering study in 2001 [123]. They analysed markers of activation in human umbilical vein endothelial cells (HUVECs) and the expression of adhesion molecules by U937 monocytes after exposing them to IgM anti-β2GPI antibodies, followed by the administration of fluvastatin or simvastatin. Their experiments revealed a dose-dependent reduction in the expression of E-selectin and ICAM by U937 monocytes, which decreased their adhesion to the HUVECs. These effects were similar for both fluvastatin and simvastatin and were mediated by the inhibition of NF-κB.

The protective role of fluvastatin in APS models was later confirmed by two separate studies conducted by Ferrara et al. In the first study, using CD-1 mice exposed to IgG aPL from patients with APS, fluvastatin administration was associated with smaller thrombi, a reduced number of adherent leukocytes, and decreased levels of ICAM-1 compared with animals treated with a placebo [124]. In the second study, involving HUVECs activated by IgG aPL, they demonstrated a dose-dependent reduction in TF expression [125].

The antithrombotic effects of statins were further corroborated by subsequent studies. Belizna et al. demonstrated that rosuvastatin administration in CD-1 mice treated with monoclonal aPL derived from lupus-like murine models recovered the dilation of mesenteric arteries through NO-mediated mechanisms [128].

Jajoria et al., in a study of 9 patients with APS treated with 40 mg of fluvastatin a day for 1 month, observed reduced levels of vascular endothelial growth factor (VEGF), TF, and TNF-α [129]. Lopez-Pedreda et al., in a study involving 42 patients with APS treated with 20 mg of fluvastatin a day for 1 month, found significant inhibition of monocyte protein activator receptors 1 and 2, as well as reduced expressions of VEGF and Flt1, mediated by the inhibition of p38 MAPK and NFκB/Rel DNA-binding activity [130].

These findings were further confirmed by Wang et al., who studied human THP-1 monocytes (derived from an acute monocytic leukaemia patient) stimulated with LPS and anti-β2GP1/β2GP1 complexes. They demonstrated that reduced TF expression in monocytes was mediated by the inhibitory effects of statins on NF-κB [131].

In 2015, Erkan et al. investigated the potential beneficial effects of statins in 24 aPL-positive patients, including 8 with primary APS, 5 primary aPL carriers, 7 with SLE-related APS, and 4 SLE aPL carriers. These patients were prescribed 40 mg of fluvastatin day for 3 months. Following treatment, the levels of IL-6, IL-1β, VEGF, TNF-α, IFN-α, IP-10, sCD40L, and TF significantly decreased, demonstrating that pro-inflammatory and pro-thrombotic biomarkers upregulated in persistently aPL-positive patients can be reversibly reduced by fluvastatin [133].

This concept was further investigated by van den Hoogen et al., who studied 47 SLE patients, 28 of whom had SLE-related APS and 24 of whom had primary APS. They observed decreased expressions of pro-inflammatory and pro-thrombotic proteins regulated by interferon I in statin users [134]. Similarly, Kotyla et al., in a study of 15 SLE aPL carriers prescribed 20 mg of simvastatin a day for 28 days, reported decreased concentrations of IL-6, CRP, CAM, and P-selectin, as well as reduced levels of aCL IgG and β2GP1 IgG titres [135].

Conversely, some studies have reported potential detrimental or no effects of statins in APS. Dunoyer-Geindre et al. showed that the incubation of HUVEC with IgG anti-β2GP1 increased the expression of VCAM-1, E-selectin, TF, and MCP-1, and that exposure to fluvastatin further increased the expression of these proteins [126]. Musial et al., utilising blood samples from 45 patients with LAC and/or markedly elevated levels of aCL IgG antibodies (>40 GPL), found that cerivastatin administration, despite the expected decrease in cholesterol, was not associated with a reduction in thrombin formation or a decrease in inflammatory markers [127]. This finding was confirmed in 11 patients with APS (4 with primary APS) treated with simvastatin [127]. Additionally, Willis et al., in a study of 21 patients with SLE—64% positive for aCL IgG, 13% for aCL IgM, 65% for β2GP1 IgG, and 45% for β2GP1 IgM—found no significant differences in IL-6, IL-8, VEGF, sCD40L, IL-1β, TNF-α, CRP, ICAM-1, VCAM-1, aCL IgG, or aCL IgM levels, nor in disease activity as measured by the SLAM-R score, following statin treatment [132].

Finally, Mazurek et al., in a separate study of 18 patients with SLE (only 2 with high aPL titres), found that 20 mg of simvastatin a day for 28 days resulted in decreased CRP levels only, with no changes in ICAM-1, vWF, or aCL IgG and β2GP1 IgG titres [136].

The reasons behind the lack of anti-inflammatory effects of statins in these latter studies remain largely unclear. The use of different models with different types of statins at varying dosages may partially explain these discrepancies. However, this further highlights the need for clinical studies aimed at identifying the optimal statin class and dosage based on the baseline risk of recurrent thrombosis.

Only two retrospective observational studies have investigated the risk of thrombosis in patients with APS during follow-up in relation to statin use (Table 2).

The first study evaluated the risk of a first thrombotic event in a population of 80 SLE patients with aPL positivity, of whom 23 (28.8%) were on statins, over a mean follow-up period of 69 months. Cox regression analysis, adjusted for age and aCL titre, showed that statin treatment was associated with a reduced risk of first thrombosis (HR of 0.12, 95% CI of 0.01–0.98). However, no statistically significant differences were observed when analysing only patients with medium to high aPL titres [137].

The second study focused on the risk of recurrent thrombosis in a cohort of 184 patients with thrombotic APS, of whom 103 (55.6%) were on statins. After a median follow-up of 48.5 months, and adjusting for OAC, antiplatelet, and hydroxychloroquine use, a reduced risk of recurrent thrombosis was observed in statin users (HR of 0.28, 95% CI of 0.10–0.76) [138].

## 8. Clinical Considerations and Future Perspectives

Emerging evidence over recent years suggests that the thrombotic risk in patients with APS is dynamic and evolves through different mechanisms during its natural course. For example, the higher prevalence of VTE as an initial presentation indicates that thromboembolic events due to the direct activation of the coagulation cascade is likely the pivotal mechanism in the early phases. However, the progressive increase in the incidence of ATE over time, even in patients already on OACs, has led to the hypothesis that, in later stages, thrombotic risk is more strongly driven by atherosclerotic processes, which are not effectively counteracted by OAC. Indeed, enhanced atherosclerosis has been reported in patients with APS from its early stages [70,139]. This can progress due to the pro-inflammatory state associated with APS, eventually leading to the onset of cardiovascular events in the later stages [140]. In this context, statins may represent a valuable therapeutic tool. They not only mitigate the high risk of VTE, ATE, and atherosclerotic events but could also address other clinical manifestations linked to a pro-inflammatory state and increased oxidative stress, which are indirectly associated with a possible late increased thrombotic risk.

Thrombocytopenia is a common clinical finding in patients with APS, resulting from the production of autoantibodies, increased platelet consumption, and reduced production of platelet precursors, often influenced by elevated ROS levels [83]. Paradoxically, in these patients, thrombocytopenia is associated with an increased risk of recurrent thrombosis, likely due to antibody-mediated platelet activation [141,142,143]. In steroid-resistant immune thrombocytopenia, statins have been shown to support megakaryocytopoiesis [144], potentially offering an additional mechanism to counteract the pro-thrombotic state in patients with APS.

Diastolic dysfunction and heart failure with preserved ejection fraction (HFpEF) are frequently reported in APS patients [145,146]. These conditions are interrelated, as diastolic dysfunction often precedes the development of HFpEF, which subsequently increases the risk of cardiovascular events [147]. In non-autoimmune contexts, statins have been linked to improved cardiac endothelial function, reducing the likelihood of HFpEF [148]. Additionally, in patients with HFpEF, statin treatment has been associated with better survival rates and a reduced risk of cardiovascular events [149], potentially offering additional benefits for individuals with APS.

One recent study showed that low-grade endotoxemia is common in patients with APS and is associated with an increased risk of recurrent thrombosis [76]. There is evidence that statins have a beneficial impact on gut permeability by upregulating the expression of intestinal adhesion proteins and inhibiting the detrimental effects of low-grade endotoxemia [150,151].

While statins appear to be an ideal complement to OACs for addressing residual thrombotic risk in APS patients, evidence supporting their use in this specific context remains limited. Most clinical studies evaluating the role of statins in reducing thrombosis or cardiovascular events have been conducted in patients without autoimmune diseases or in individuals with SLE—a condition sometimes associated with aPL positivity or defined APS. However, including SLE patients without specific analyses based on the aPL profile introduces confounding factors, limiting the applicability of these findings to APS patients. Furthermore, current evidence on APS primarily stems from pre-clinical studies, with clinical data largely derived from retrospective observational research. The potential for unmeasured biases in these studies necessitates cautious interpretation of their findings when formulating therapeutic recommendations.

Additionally, it is worth noting that some reports in non-autoimmune settings have suggested that statin administration may be associated with increased aPL titres [152] or elevated PCSK9 (proprotein convertase subtilisin/kexin type 9) levels [153]. Both factors have been linked to a higher thrombotic risk in APS [13,154], underscoring the need for further studies to better understand these potential associations.

In conclusion, future RCTs specifically designed to evaluate the superiority of alternative antithrombotic strategies over the traditional moderate-intensity VKA regimen are essential. Notably, studies investigating the potential role of statins as an adjunct to OACs should consider factors such as dosage variations, differences between statin classes (e.g., hydrophilic versus lipophilic), the thrombotic phenotype (e.g., ATE or VTE as the initial presentation), the presence of coexisting systemic autoimmune diseases, and the aPL profile.

## Figures and Tables

**Table 1:** Beneficial effects of statins in preclinical studies on antiphospholipid syndrome.

Year of Publication/First Author	Model	Type of Statin	Type of aPL/APS	Main Results
2001 Meroni [123]	HUVEC U937 monocyte	Fluvastatin Simvastatin	Polyclonal affinity-purified IgG Monoclonal IgM anti-β2GPI antibodies Human recombinant IL-1β, TNFα, or LPS	Fluvastatin reduced, in a concentration-dependent manner, the adhesion of HUVECs and monocytes and the expressions of E-selectin and ICAM-1 induced by anti-β 2GPI antibodies, as well as by cytokines or LPS These effects were mediated by the inhibition of NFκB Simvastatin displayed similar effects, but to a lesser extent than fluvastatin
2003 Ferrara [124]	CD1 mice	Fluvastatin	IgG from patients with the APS	Mice treated with IgG-APS and fluvastatin showed significantly smaller thrombi, a reduced number of adherent leukocytes, and decreased levels of ICAM-1 compared with IgG-APS animals treated with placebo
2004 Ferrara [125]	HUVEC	Fluvastatin	IgG from patients with the antiphospholipid syndrome (IgG-APS)	Fluvastatin inhibited the effects of IgG-APS on tissue factor in a dose-dependent manner
2005 Dunoyer-Geindre [126]	HUVEC	Fluvastatin	IgG anti-β2GP1 from six patients with APS	Incubation of HUVECs with patient IgG anti-β2GP1 increased the expressions of VCAM-1, E-selectin, TF, and MCP-1. Prior treatment of HUVECs with fluvastatin further increased the expression of these proteins
2005 Musial [127]	45 APS patients received cerivastatin or placebo in a double-blind, randomised fashion (2:1 design) After withdrawal of cerivastatin from the market, 11 additional patients were given simvastatin in an open-label study	Cerivastatin, 0.4 mg/day for 28 days Simvastatin, 40 mg/day for 28 days	Lupus anticoagulant and/or markedly increased levels of aCL IgG antibodies (> 40 GPL)	After statin treatment, despite the expected decrease in total and LDL cholesterol, there was no reduction in thrombin formation at the site of microvascular injury, nor was there any lowering of inflammatory markers
2008 Belizna [128]	CD1 mice	Rosuvastatin	aPL monoclonal antibodies derived from male (BXSB × NZW) F1 mice with a lupus-like disease	Antiphospholipid monoclonal antibodies reduced the response to acetylcholine of mesenteric arteries This effect was mediated by a reduced production of NO that was prevented by the administration of statins
2009 Jajoria [129]	Blood samples from 9 patients	Fluvastatin, 40 mg/day for 1 month	APS (unspecified)	Fluvastatin significantly reduced the levels of vascular endothelial growth factor (VEGF), TF, and TNF-α
2011 López-Pedrera [130]	Blood samples from 42 patients	Fluvastatin, 20 mg/day for 1 month	APS (unspecified)	Monocytes showed a significant inhibition of TF, protein activator receptors 1 and 2, VEGF, and Flt1 expression, which was related to the inhibition of p38 MAPK NFκB/Rel DNA-binding activity Reduced levels of proteins involved in thrombotic development (annexin II, RhoA, and protein disulphide isomerase) after fluvastatin administration
2013 Wang [131]	Human monocytes THP-1 (derived from an acute monocytic leukaemia patient)	Fluvastatin	Monocytes THP-1 were treated with fluvastatin, LPS, and anti-β2GP1/β2GP1 complexes	Fluvastatin interfered with the expression of the NF-kB signalling transduction pathway, thereby decreasing the expressions of TF and TNF-α
2014 Willis [132]	21 patients with SLE	Any statins	64% were positive for aCL IgG, 13% for aCL IgM, 65% for β2GP1 IgG, and 45% for β2GP1 IgM	No significant differences were found after statin treatment for Il-6, IL-8, VEGF, sCD40L, IL-1β, TNF-α, CRP, ICAM-1, VCAM-1, aCL IgG, aCL IgM, or disease activity (SLAM-R score)
2015 Erkan [133]	Blood samples from 24 aPL positive patients	Fluvastatin, 40 mg/day for 3 months	8 primary APS, 7 SLE + APS, 5 primary aPL carriers, and 4 SLE aPL carriers	The levels of IL-6, IL-1β, VEGF, TNF-α, IFN-α, IP-10, sCD40L, and TF significantly decreased with fluvastatin
2016 van den Hoogen [134]	Monocytes from blood samples of 99 patients	Any statins	47 SLE patients, 28 SLE-related APS, and 24 primary APS	Monocytes from patients prescribed statins showed lower expressions of pro-inflammatory proteins regulated by interferon-I
2018 Kotyla [135]	Blood samples from 15 SLE patients	Simvastatin, 20 mg/day for 28 days	aPL carriers	Simvastatin administration resulted in a significant reduction of aCL IgG, β2GP1 IgG, IL-6, CRP, ICAM-1, and p-Selectin, but not IL-1β or TNF-α
2022 Mazurek [136]	18 SLE patients	Atorvastatin, 40 mg/day for 1 year	2 patients with aCL or anti-β2GP1 IgG ≥ 40 GPL or SGU	Atorvastatin was associated with a decrease in CRP levels only, whereas ICAM-1, vWF, and aPL remained unchanged

Legend: aCL: anti-cardiolipin antibodies, APS: antiphospholipid syndrome, β2GPI: Beta 2 Glycoprotein I, CRP: C-reactive protein, Flt1: vascular endothelial growth factor receptor 1, HUVEC: human umbilical vein endothelial cell, ICAM: intercellular adhesion molecule, IFN-α: alpha interferon, IL-1β: Interleukin-1 beta, LPS: lipopolysaccharide, MAPK: mitogen-activated protein kinase, MCP-1: Monocyte Chemoattractant Protein-1, NFκB: nuclear factor κB, NO: nitric oxide, TF: tissue factor, VCAM-1: vascular cell adhesion protein 1, VEGF: vascular endothelial growth factor, SLAM-R: Systemic Lupus Activity Measure, Revised SLE: systemic lupus erythematosus, TNF-α: tumour necrosis factor alfa, vWF: Von Willebrand factor.

**Table 2:** Beneficial effects of statins in clinical studies on antiphospholipid syndrome.

Year of Publication and First Author	Design	Number of Patients Included/Mean Follow up	Type of Statin	Main Results
2017 Watanabe [137]	Retrospective observational	80 SLE patients positive for aPL (without previous thrombosis), of whom 23 were on statins 69 months	Any statins	After adjusting for age and aCL titre, statin treatment was associated with reduced risk of first thrombosis (HR of 0.12, 95% CI of 0.01–0.98) When considering only those with high aPL titre, the protective effect was not statistically significant (HR of 0.16, 95% CI of 0.02–1.30)
2022 Kwon [138]	Retrospective observational	184 patients with thrombotic APS, of whom 103 were on statins 48.5 months	Any statins	After adjusting for the use of anticoagulants, antiplatelets, and hydroxychloroquine, statins were associated with a reduced risk of recurrent thrombosis (HR of 0.28, 95% CI of 0.10, 0.76)

aPL: anti-phospholipid antibodies, HR: hazard ratio, CI: confidence interval.

## Data Availability

No new data were created or analyzed in this study.
